# Supplementary material for: The value of combining individual and small area sociodemographic data for assessing and handling selective participation in cohort studies: Evidence from the Swedish CardioPulmonary bioImage Study
Source: PLoS One. 2022 Mar 8;17(3):e0265088. doi: 10.1371/journal.pone.0265088 (PMC8903292; doi:10.1371/journal.pone.0265088)
Supplement: S1 Appendix — (DOCX) [file pone.0265088.s001.docx]

**Supplementary Appendix**

This document contains supplementary tables, figures and mathematical derivations for Bonander et al. *"* *The value of combining individual and small area sociodemographic data for assessing and handling selective participation in cohort studies: evidence from the Swedish CardioPulmonary bioImage Study”*

Table of Contents

[Supplementary figures 4](#_Toc76115093)

[Supplementary tables 10](#_Toc76115094)

[Mathematical derivations 14](#_Toc76115095)

[Method for inferring the characteristics of non-participants in Table 1 14](#_Toc76115096)

[Method for calculating the AUC for classification of participants and non-participants in data where non-participants are not observed 15](#_Toc76115097)

**List of supplementary figures (with page numbers)**

[**Figure S1.** Map of Sweden highlighting the recruitment areas for the Swedish CardioPulmonary bioImage Study (SCAPIS), with maps of estimated neighborhood-level participation rates per site based on the combined participation model with individual-level and area-level socioeconomic factors (and their interactions). The bottom subplot shows the inverse correlation between estimated participation probabilities and area-level economic status using Malmö as an illustrative example. 4](#_Toc75160481)

[**Figure S2.** Participation probabilities based on logistic regression models with individual-level sociodemographics (a), neighborhood-level sociodemographics (b), and both (c), estimated for participants and non-participants in the Swedish CardioPulmonary bioImage Study (SCAPIS). 5](#_Toc75160482)

[**Figure S3.** Density distributions of propensity scores for participation in SCAPIS, within each stratum of the individual-level characteristics and site, based on a model with individual-level and neighborhood-level characteristics (and their interactions). 6](#_Toc75160483)

[**Figure S4**. Standardized difference between the unweighted SCAPIS participants and weighted SCAPIS participants in the age group 50-54 years standardized to match the target population on individual and neighborhood-level sociodemographic characteristics, with reference lines at -0.10, -0.05, 0.05 and 0.10 to highlight potentially meaningful differences. 7](#_Toc75160484)

[**Figure S5.** Standardized difference between the unweighted SCAPIS participants and weighted SCAPIS participants in the age group 55-59 years standardized to match the target population on individual and neighborhood-level sociodemographic characteristics, with reference lines at -0.10, -0.05, 0.05 and 0.10 to highlight potentially meaningful differences. 8](#_Toc75160485)

[**Figure S6.** Standardized difference between the unweighted SCAPIS participants and weighted SCAPIS participants in the age group 60-64 years standardized to match the target population on individual and neighborhood-level sociodemographic characteristics, with reference lines at -0.10, -0.05, 0.05 and 0.10 to highlight potentially meaningful differences. 9](#_Toc75160486)

**List of supplementary tables (with page numbers)**

[**Table S1.** Sociodemographic characteristics of the participants in the Swedish CardioPulmonary bioImage Study (SCAPIS), a random sample of its target population, after weighting the participants to match the target population on individual-level sociodemographic characteristics. 10](#_Toc75160477)

[**Table S2.** Sociodemographic characteristics of the participants in the Swedish CardioPulmonary bioImage Study (SCAPIS), a random sample of its target population, after weighting the participants to match the target population on neighborhood-level sociodemographic characteristics and site. 11](#_Toc75160478)

[**Table S3.** Sociodemographic characteristics of the participants in the Swedish CardioPulmonary bioImage Study (SCAPIS), a random sample of its target population, after weighting the participants to match the target population on individual-level sociodemographic characteristics, neighborhood-level sociodemographic characteristics and site. 12](#_Toc75160479)

[**Table S4.** Distribution of sociodemographic, metabolic and behavioral predictors of cardiovascular disease among participants in the Swedish CardioPulmonary bioImage Study (SCAPIS), with inferred distributions for non-participants and the target population for the study. 13](#_Toc75160480)

# Supplementary figures


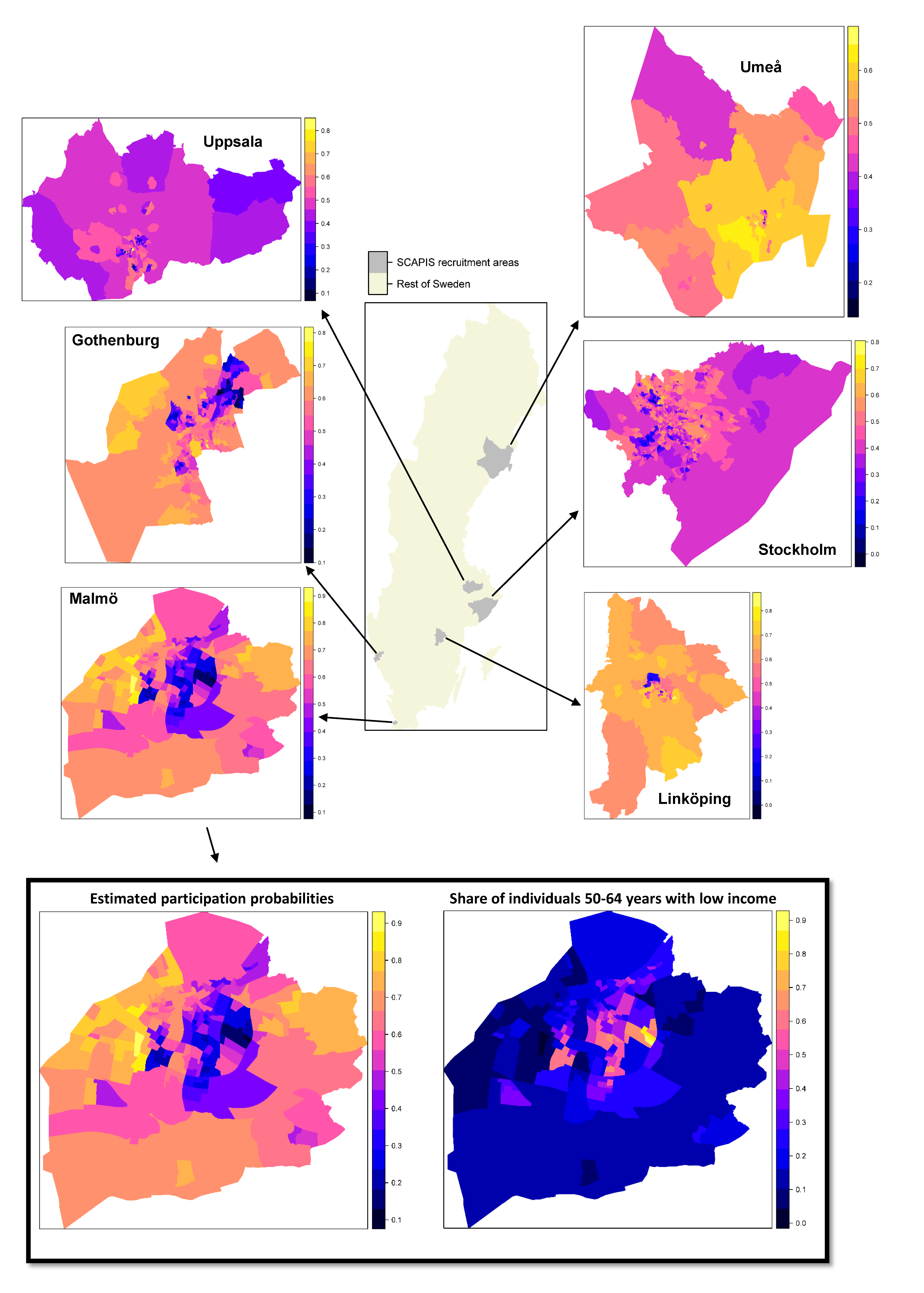


**Figure S1.** Map of Sweden highlighting the recruitment areas for the Swedish CardioPulmonary bioImage Study (SCAPIS), with maps of estimated neighborhood-level participation rates per site based on the combined participation model with individual-level and area-level socioeconomic factors (and their interactions). The bottom subplot shows the inverse correlation between estimated participation probabilities and area-level economic status using Malmö as an illustrative example.


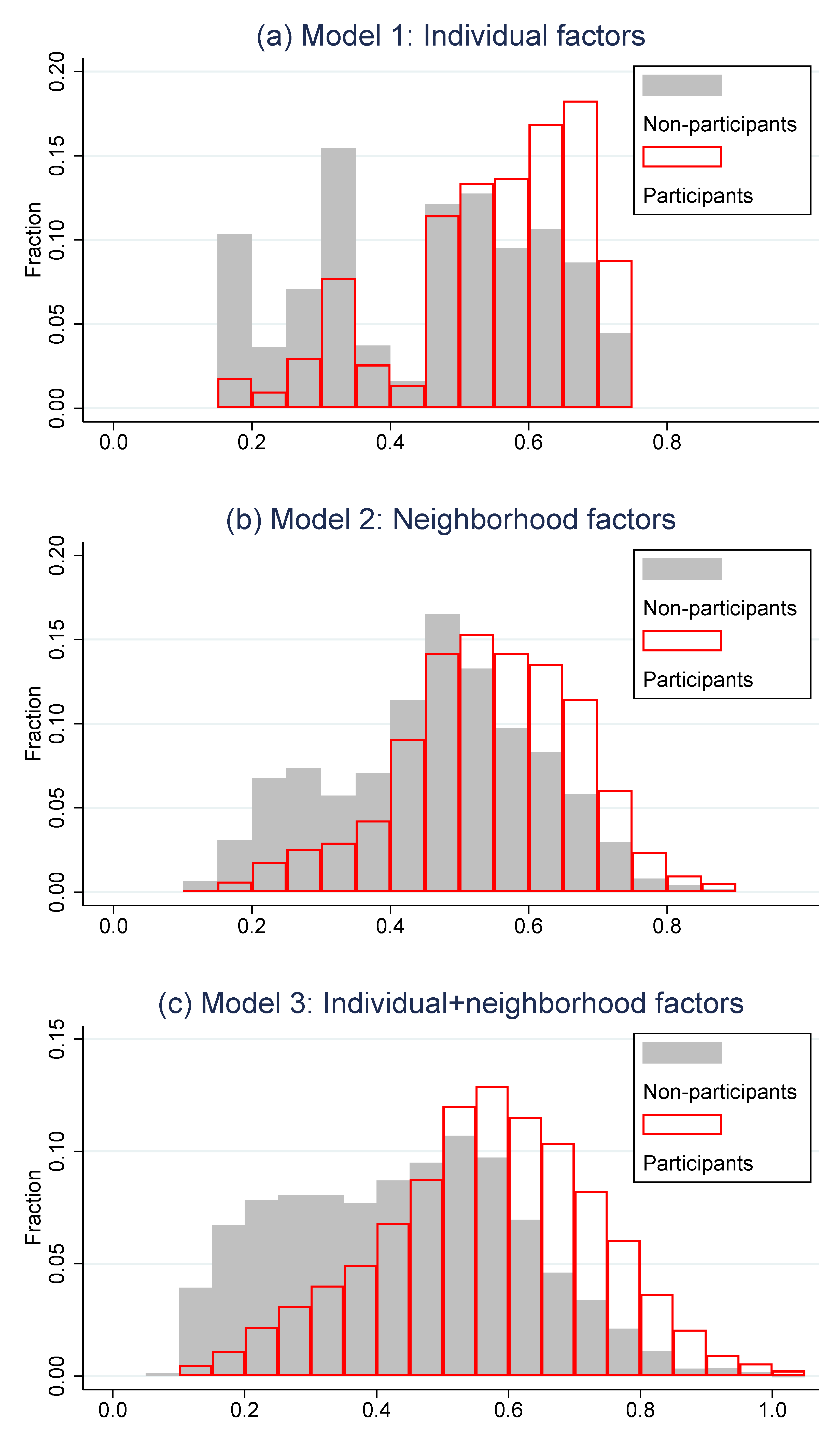


**Figure S2.** Participation probabilities based on logistic regression models with individual-level sociodemographics (a), neighborhood-level sociodemographics (b), and both (c), estimated for participants and non-participants in the Swedish CardioPulmonary bioImage Study (SCAPIS).

^
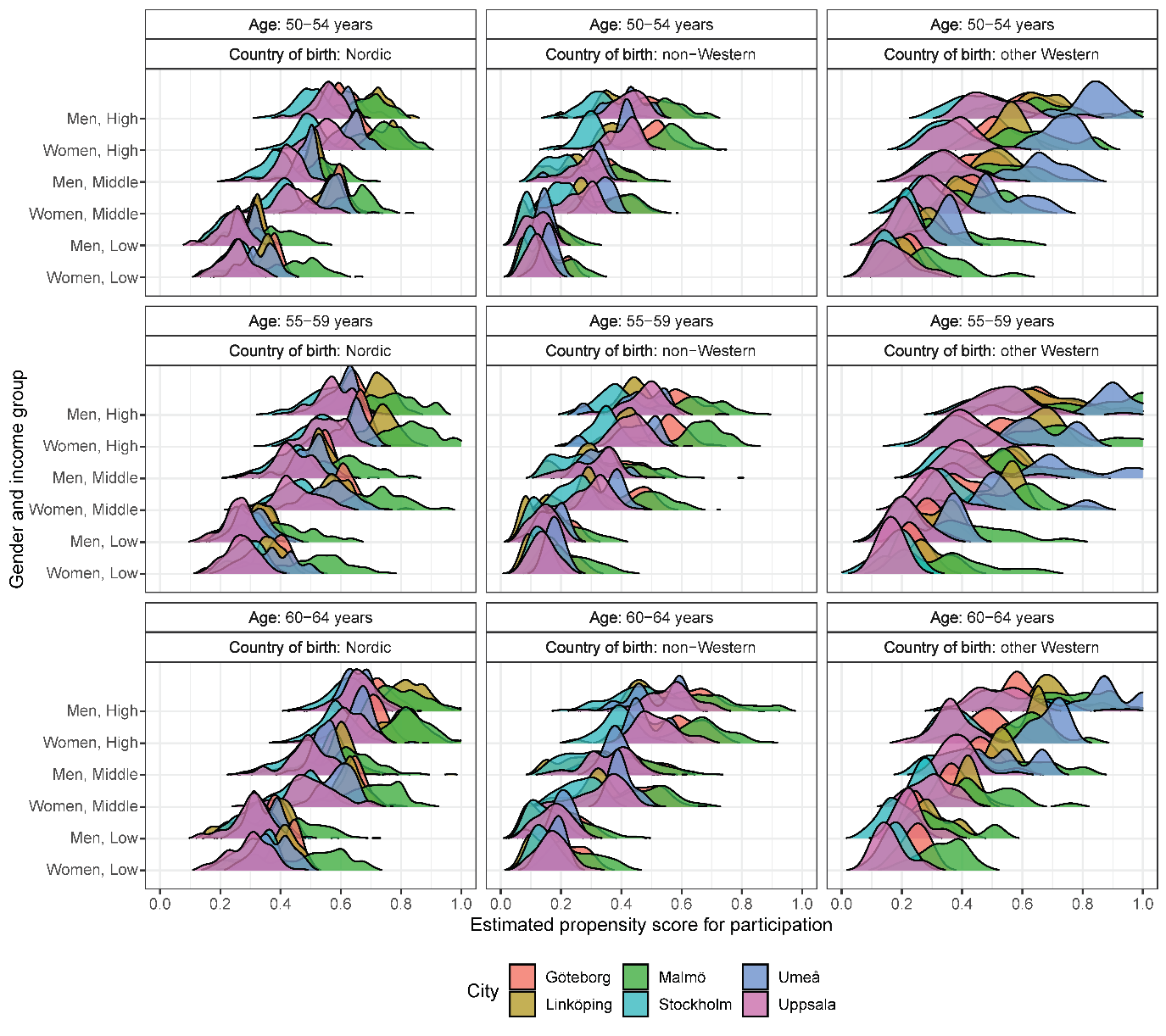
^

**Figure S3.** Density distributions of propensity scores for participation in SCAPIS, within each stratum of the individual-level characteristics and site, based on a model with individual-level and neighborhood-level characteristics (and their interactions).


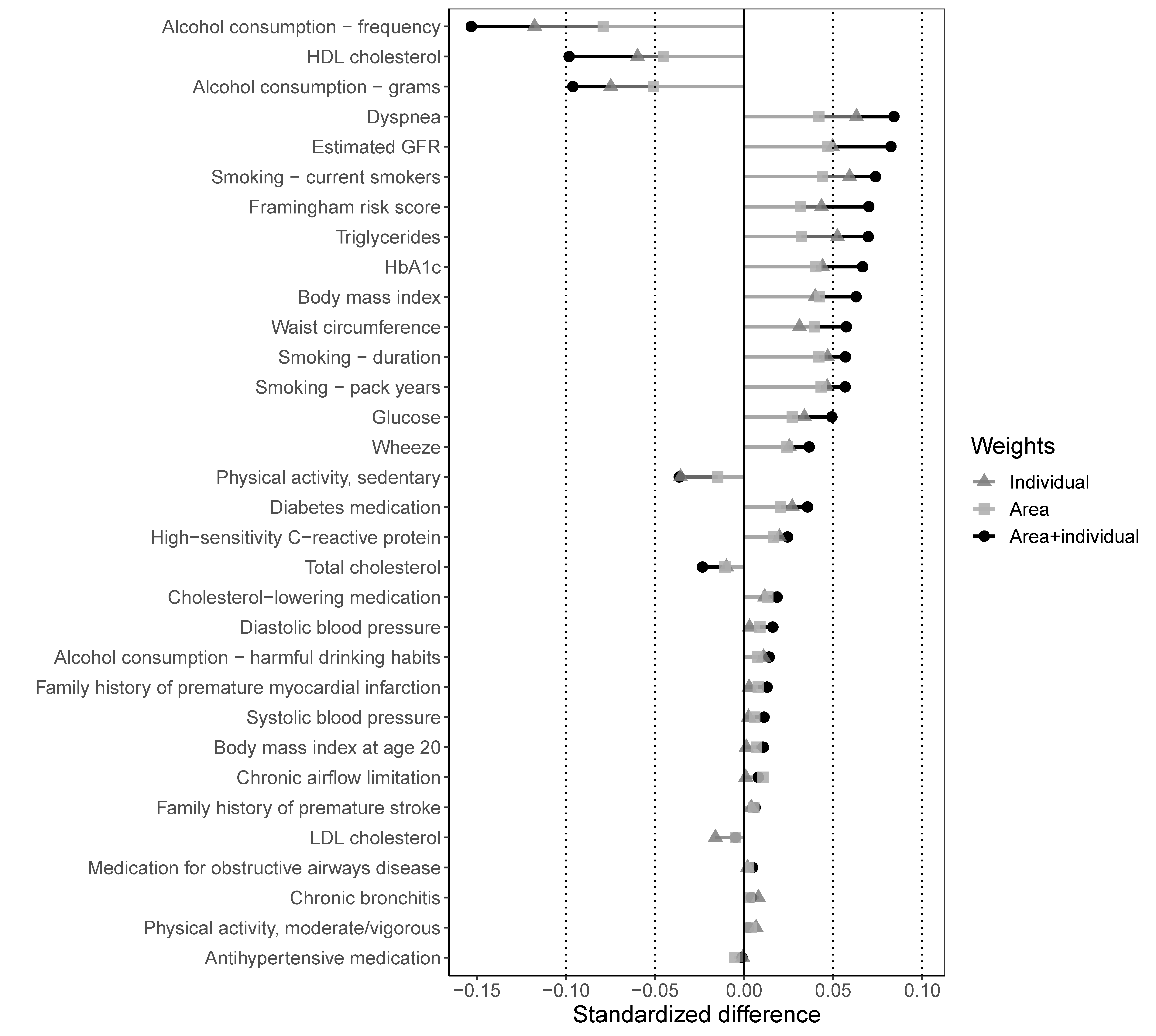


**Figure S4**. Standardized difference between the unweighted SCAPIS participants and weighted SCAPIS participants in the age group 50-54 years standardized to match the target population on individual and neighborhood-level sociodemographic characteristics, with reference lines at -0.10, -0.05, 0.05 and 0.10 to highlight potentially meaningful differences.


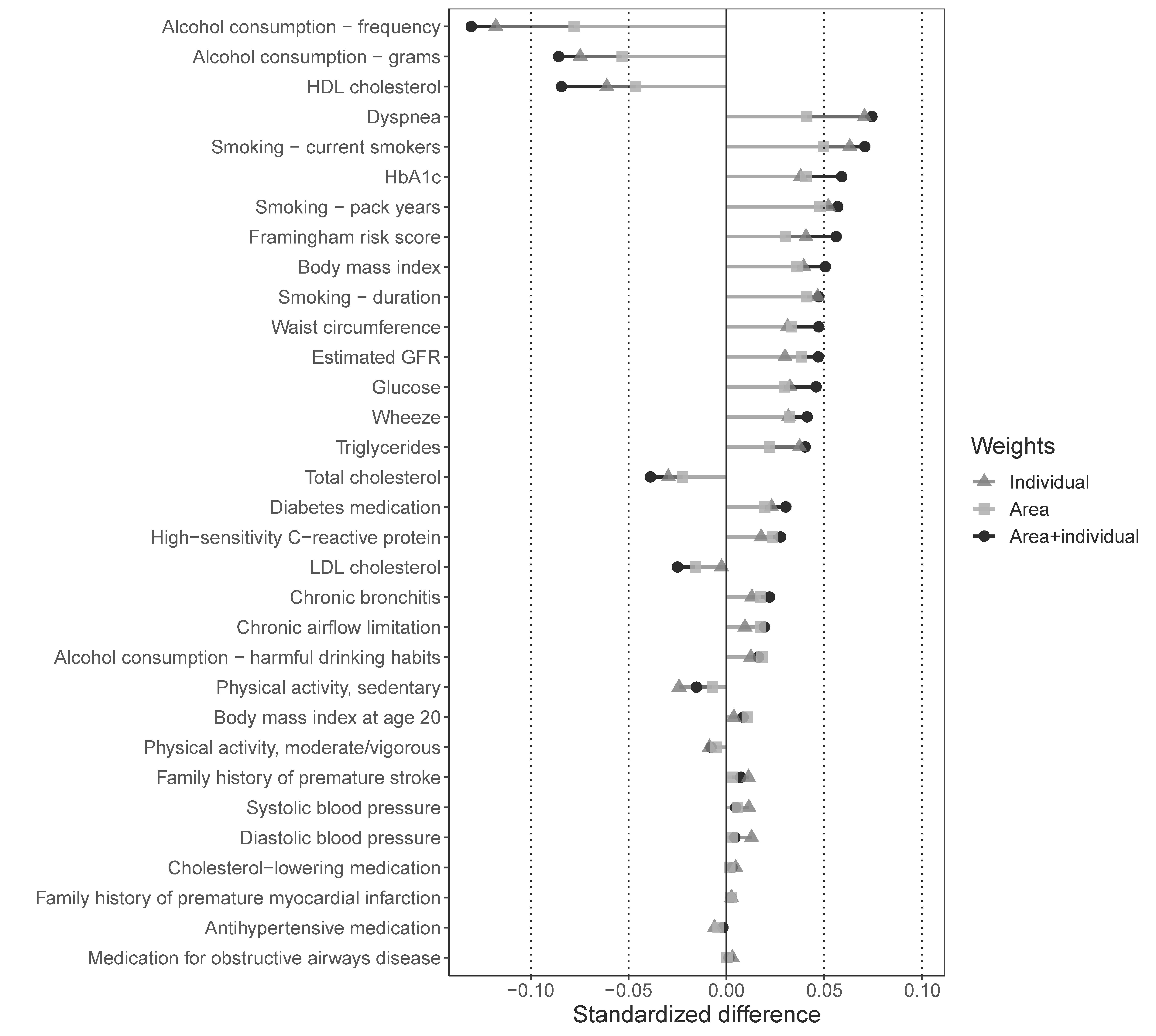


**Figure S5.** Standardized difference between the unweighted SCAPIS participants and weighted SCAPIS participants in the age group 55-59 years standardized to match the target population on individual and neighborhood-level sociodemographic characteristics, with reference lines at -0.10, -0.05, 0.05 and 0.10 to highlight potentially meaningful differences.


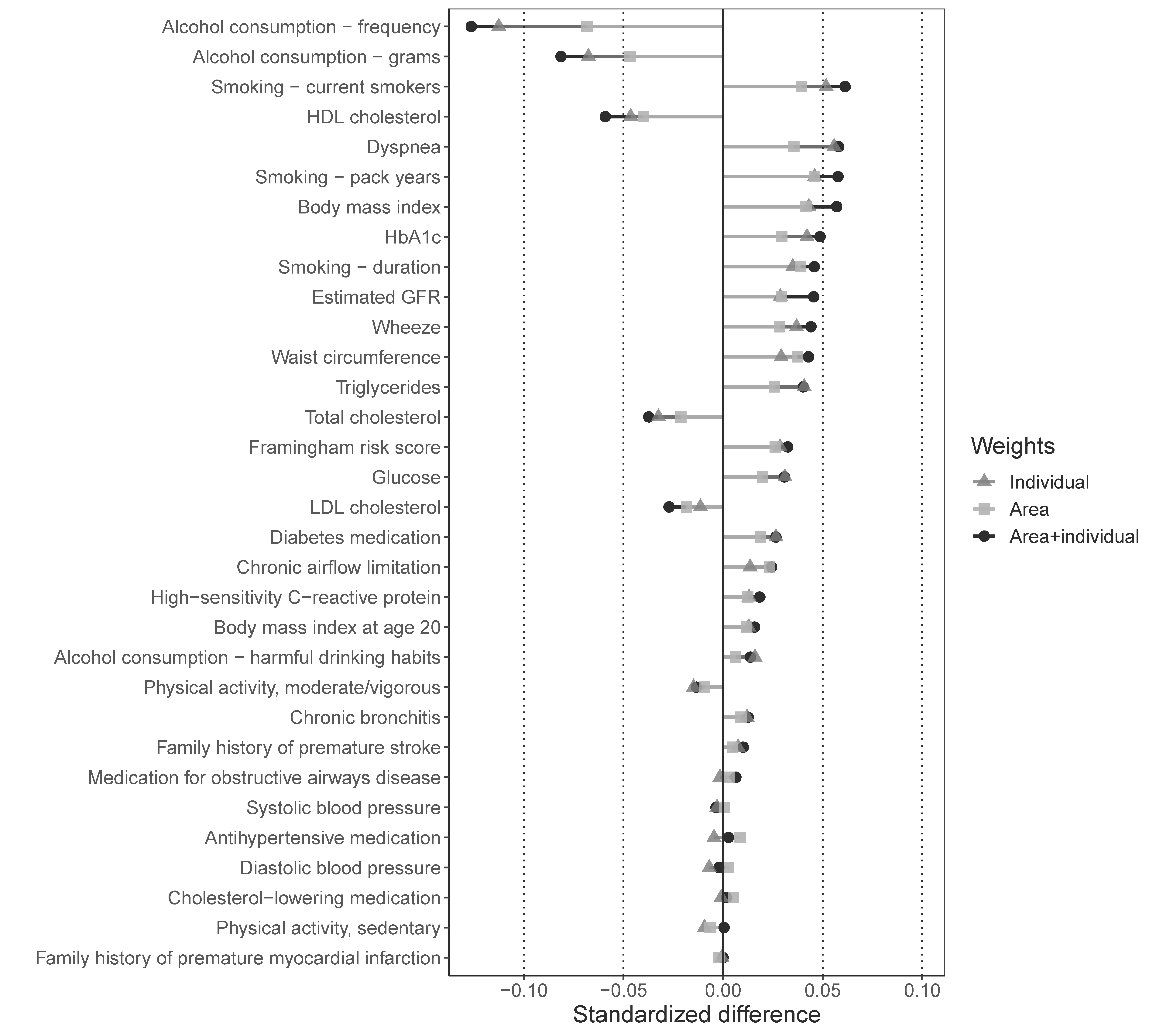


**Figure S6.** Standardized difference between the unweighted SCAPIS participants and weighted SCAPIS participants in the age group 60-64 years standardized to match the target population on individual and neighborhood-level sociodemographic characteristics, with reference lines at -0.10, -0.05, 0.05 and 0.10 to highlight potentially meaningful differences.

# Supplementary tables

**Table S1.** Sociodemographic characteristics of the participants in the Swedish CardioPulmonary bioImage Study (SCAPIS), a random sample of its target population, after weighting the participants to match the target population on individual-level sociodemographic characteristics.

| Characteristic | Participants,  unweighted | Participants,  weighted | Target population  (sample) | Absolute  SMD^a^ |
| --- | --- | --- | --- | --- |
| N | 30,154 | 31,067.3 | 59,909.0 |  |
| Men, n (%) | 14,646 (48.6) | 15,423.8 (49.6) | 29,822.0 (49.8) | 0.003 |
| Age group, n (%) |  |  |  | 0.007 |
| 50-54 y | 10,049 (33.3) | 11,309.6 (36.4) | 22,000.0 (36.7) |  |
| 55-59 y | 9,980 (33.1) | 10,251.3 (33.0) | 19,693.0 (32.9) |  |
| 60-64 y | 10,125 (33.6) | 9,506.4 (30.6) | 18,216.0 (30.4) |  |
| Income group, n (%) |  |  |  | 0.009 |
| High | 16,927 (56.1) | 14,074.3 (45.3) | 26,980.0 (45.0) |  |
| Middle | 10,630 (35.3) | 11,760.2 (37.9) | 22,636.0 (37.8) |  |
| Low | 2,597 (8.6) | 5,232.8 (16.8) | 10,293.0 (17.2) |  |
| Country of birth, n (%) |  |  |  | 0.012 |
| Nordic | 26,074 (86.5) | 24,191.4 (77.9) | 46,367.0 (77.4) |  |
| Other western | 601 (2.0) | 727.8 (2.3) | 1,396.0 (2.3) |  |
| Non-western | 3,479 (11.5) | 6,148.2 (19.8) | 12,146.0 (20.3) |  |
| Site, n (%) |  |  |  | 0.135 |
| Gothenburg | 6,266 (20.8) | 6,623.9 (21.3) | 12,109.0 (20.2) |  |
| Linköping | 5,056 (16.8) | 4,806.5 (15.5) | 8,721.0 (14.6) |  |
| Malmö | 6,251 (20.7) | 7,260.3 (23.4) | 11,763.0 (19.6) |  |
| Stockholm | 5,038 (16.7) | 5,003.9 (16.1) | 11,950.0 (19.9) |  |
| Umeå | 2,507 (8.3) | 2,410.7 (7.8) | 4,603.0 (7.7) |  |
| Uppsala | 5,036 (16.7) | 4,962.0 (16.0) | 10,763.0 (18.0) |  |
| Neighborhood-level characteristics (mean (SD)) |  |  |  |  |
| % low income households, ages 50-64 | 13.87 (11.32) | 15.72 (13.03) | 17.04 (14.36) | 0.096 |
| % middle income households, ages 50-64 | 36.74 (9.85) | 37.58 (9.86) | 37.92 (9.93) | 0.035 |
| % high income households, ages 50-64 | 49.39 (18.06) | 46.70 (19.25) | 45.03 (20.24) | 0.084 |
| % of Nordic origin, ages 50-64 | 80.93 (16.95) | 78.24 (19.30) | 76.35 (21.31) | 0.093 |
| % of other Western origin, ages 50-64 | 2.32 (1.34) | 2.33 (1.36) | 2.37 (1.41) | 0.028 |
| % of non-Western origin, ages 50-64 | 16.75 (16.76) | 19.43 (19.15) | 21.29 (21.19) | 0.092 |
| % with university education, ages 50-64 | 45.24 (15.29) | 43.81 (15.51) | 42.75 (15.85) | 0.067 |
| % unemployed working-age individuals | 20.38 (9.44) | 21.70 (10.45) | 22.54 (11.28) | 0.078 |
| % single parent households | 6.87 (2.63) | 7.13 (2.84) | 7.41 (3.04) | 0.094 |
| % rental housing | 28.84 (28.82) | 31.73 (30.57) | 33.92 (32.06) | 0.070 |

^a Absolute standardized difference between weighted participants and the target population sample.^

**Table S2.** Sociodemographic characteristics of the participants in the Swedish CardioPulmonary bioImage Study (SCAPIS), a random sample of its target population, after weighting the participants to match the target population on neighborhood-level sociodemographic characteristics and site.

| Characteristic | Participants,  unweighted | Participants,  weighted | Target population  (sample) | Absolute  SMD^a^ |
| --- | --- | --- | --- | --- |
| N | 30,154 | 30,925.6 | 59,909.0 |  |
| Men, n (%) | 14,646 (48.6) | 15,033.2 (48.6) | 29,822.0 (49.8) | 0.023 |
| Age group, n (%) |  |  |  | 0.078 |
| 50-54 y | 10,049 (33.3) | 10,315.5 (33.4) | 22,000.0 (36.7) |  |
| 55-59 y | 9,980 (33.1) | 10,277.4 (33.2) | 19,693.0 (32.9) |  |
| 60-64 y | 10,125 (33.6) | 10,332.8 (33.4) | 18,216.0 (30.4) |  |
| Income group, n (%) |  |  |  | 0.201 |
| High | 16,927 (56.1) | 16,123.4 (52.1) | 26,980.0 (45.0) |  |
| Middle | 10,630 (35.3) | 11,476.4 (37.1) | 22,636.0 (37.8) |  |
| Low | 2,597 (8.6) | 3,325.8 (10.8) | 10,293.0 (17.2) |  |
| Country of birth, n (%) |  |  |  | 0.145 |
| Nordic | 26,074 (86.5) | 25,675.4 (83.0) | 46,367.0 (77.4) |  |
| Other western | 601 (2.0) | 660.1 (2.1) | 1,396.0 (2.3) |  |
| Non-western | 3,479 (11.5) | 4,590.1 (14.8) | 12,146.0 (20.3) |  |
| Site, n (%) |  |  |  | 0.005 |
| Gothenburg | 6,266 (20.8) | 6,305.1 (20.4) | 12,109.0 (20.2) |  |
| Linköping | 5,056 (16.8) | 4,477.5 (14.5) | 8,721.0 (14.6) |  |
| Malmö | 6,251 (20.7) | 6,074.9 (19.6) | 11,763.0 (19.6) |  |
| Stockholm | 5,038 (16.7) | 6,143.1 (19.9) | 11,950.0 (19.9) |  |
| Umeå | 2,507 (8.3) | 2,385.1 (7.7) | 4,603.0 (7.7) |  |
| Uppsala | 5,036 (16.7) | 5,539.9 (17.9) | 10,763.0 (18.0) |  |
| Neighborhood-level characteristics (mean (SD)) |  |  |  |  |
| % low income households, ages 50-64 | 13.87 (11.32) | 16.96 (14.20) | 17.04 (14.36) | 0.006 |
| % middle income households, ages 50-64 | 36.74 (9.85) | 37.89 (9.89) | 37.92 (9.93) | 0.003 |
| % high income households, ages 50-64 | 49.39 (18.06) | 45.15 (20.07) | 45.03 (20.24) | 0.006 |
| % of Nordic origin, ages 50-64 | 80.93 (16.95) | 76.51 (20.85) | 76.35 (21.31) | 0.008 |
| % of other Western origin, ages 50-64 | 2.32 (1.34) | 2.37 (1.41) | 2.37 (1.41) | 0.003 |
| % of non-Western origin, ages 50-64 | 16.75 (16.76) | 21.12 (20.70) | 21.29 (21.19) | 0.008 |
| % with university education, ages 50-64 | 45.24 (15.29) | 42.83 (15.74) | 42.75 (15.85) | 0.005 |
| % unemployed working-age individuals | 20.38 (9.44) | 22.47 (11.17) | 22.54 (11.28) | 0.006 |
| % single parent households | 6.87 (2.63) | 7.39 (3.02) | 7.41 (3.04) | 0.005 |
| % rental housing | 28.84 (28.82) | 33.80 (31.81) | 33.92 (32.06) | 0.004 |

^a Absolute standardized difference between weighted participants and the target population sample.^

**Table S3.** Sociodemographic characteristics of the participants in the Swedish CardioPulmonary bioImage Study (SCAPIS), a random sample of its target population, after weighting the participants to match the target population on individual-level sociodemographic characteristics, neighborhood-level sociodemographic characteristics and site.

| Characteristic | Participants,  unweighted | Participants,  weighted | Target population  (sample) | Absolute  SMD^a^ |
| --- | --- | --- | --- | --- |
| N | 30,154 | 31,521.6 | 59,909.0 |  |
| Men, n (%) | 14,646 (48.6) | 15,706.6 (49.8) | 29,822.0 (49.8) | 0.001 |
| Age group, n (%) |  |  |  | 0.001 |
| 50-54 y | 10,049 (33.3) | 11,580.2 (36.7) | 22,000.0 (36.7) |  |
| 55-59 y | 9,980 (33.1) | 10,345.0 (32.8) | 19,693.0 (32.9) |  |
| 60-64 y | 10,125 (33.6) | 9,596.4 (30.4) | 18,216.0 (30.4) |  |
| Income group, n (%) |  |  |  | 0.004 |
| High | 16,927 (56.1) | 14,220.8 (45.1) | 26,980.0 (45.0) |  |
| Middle | 10,630 (35.3) | 11,927.0 (37.8) | 22,636.0 (37.8) |  |
| Low | 2,597 (8.6) | 5,373.8 (17.0) | 10,293.0 (17.2) |  |
| Country of birth, n (%) |  |  |  | 0.004 |
| Nordic | 26,074 (86.5) | 24,435.1 (77.5) | 46,367.0 (77.4) |  |
| Other western | 601 (2.0) | 741.4 (2.4) | 1,396.0 (2.3) |  |
| Non-western | 3,479 (11.5) | 6,345.2 (20.1) | 12,146.0 (20.3) |  |
| Site, n (%) |  |  |  | 0.003 |
| Gothenburg | 6,266 (20.8) | 6,401.8 (20.3) | 12,109.0 (20.2) |  |
| Linköping | 5,056 (16.8) | 4,580.3 (14.5) | 8,721.0 (14.6) |  |
| Malmö | 6,251 (20.7) | 6,168.6 (19.6) | 11,763.0 (19.6) |  |
| Stockholm | 5,038 (16.7) | 6,279.2 (19.9) | 11,950.0 (19.9) |  |
| Umeå | 2,507 (8.3) | 2,416.1 (7.7) | 4,603.0 (7.7) |  |
| Uppsala | 5,036 (16.7) | 5,675.5 (18.0) | 10,763.0 (18.0) |  |
| Neighborhood-level characteristics (mean (SD)) |  |  |  |  |
| % low income households, ages 50-64 | 13.87 (11.32) | 17.00 (14.33) | 17.04 (14.36) | 0.003 |
| % middle income households, ages 50-64 | 36.74 (9.85) | 37.92 (9.98) | 37.92 (9.93) | <0.001 |
| % high income households, ages 50-64 | 49.39 (18.06) | 45.08 (20.24) | 45.03 (20.24) | 0.002 |
| % of Nordic origin, ages 50-64 | 80.93 (16.95) | 76.39 (21.24) | 76.35 (21.31) | 0.002 |
| % of other Western origin, ages 50-64 | 2.32 (1.34) | 2.37 (1.42) | 2.37 (1.41) | 0.002 |
| % of non-Western origin, ages 50-64 | 16.75 (16.76) | 21.24 (21.10) | 21.29 (21.19) | 0.002 |
| % with university education, ages 50-64 | 45.24 (15.29) | 42.78 (15.83) | 42.75 (15.85) | 0.002 |
| % unemployed working-age individuals | 20.38 (9.44) | 22.51 (11.23) | 22.54 (11.28) | 0.003 |
| % single parent households | 6.87 (2.63) | 7.41 (3.06) | 7.41 (3.04) | <0.001 |
| % rental housing | 28.84 (28.82) | 33.90 (32.01) | 33.92 (32.06) | 0.001 |

^a Absolute standardized difference between weighted participants and the target population sample.^

**Table S4.** Distribution of sociodemographic, metabolic and behavioral predictors of cardiovascular disease among participants in the Swedish CardioPulmonary bioImage Study (SCAPIS), with inferred distributions for non-participants and the target population for the study.

| Characteristic | Participants | Target population,  inferred^a^ |
| --- | --- | --- |
| Sample size - n | 30,154 | 59,909 |
| Men - n (%) | 14,646.0 (48.6) | 29,851.6 (49.8) |
| Age group - n (%) |  |  |
| 50-54 y | 10,049.0 (33.3) | 22,009.0 (36.7) |
| 55-59 y | 9,980.0 (33.1) | 19,661.4 (32.8) |
| 60-64 y | 10,125.0 (33.6) | 18,238.6 (30.4) |
| Education, university degree - n (%) | 13,218.0 (45.1) | 24,463.7 (42.4) |
| Employed - n (%) | 24,483.0 (81.2) | 45,647.2 (76.2) |
| Body mass index at age 20 - kg/m^2^ | 21.93 (2.73) | 21.97 (2.80) |
| Body mass index - kg/m^2^ | 26.98 (4.48) | 27.24 (4.59) |
| Waist circumference - cm | 94.42 (12.95) | 94.99 (13.02) |
| Smoking status - n (%) |  |  |
| Current smoker | 3,728.0 (12.4) | 8,599.7 (14.4) |
| Former smoker | 10,602.0 (35.2) | 20,393.3 (34.0) |
| Never smoker | 14,731.0 (48.9) | 28,379.0 (47.4) |
| Unknown | 1,093.0 (3.6) | 2,537.1 (4.2) |
| Smoking - pack years | 7.45 (11.99) | 8.03 (12.62) |
| Smoking - duration in years | 11.82 (15.58) | 12.42 (15.91) |
| Alcohol consumption, frequency - n (%) |  |  |
| Once per month or less | 7,198.0 (24.7) | 17,660.4 (30.8) |
| 2-4 times per month | 11,123.0 (38.1) | 20,649.9 (36.0) |
| More than once a week | 10,865.0 (37.2) | 19,058.7 (33.2) |
| Alcohol consumption – grams per day | 7.11 (6.54) | 6.52 (6.54) |
| Physical activity, sedentary - % of active time | 53.97 (10.47) | 53.76 (10.82) |
| Physical activity, moderate or vigorous - % of active time | 6.38 (3.44) | 6.38 (3.55) |
| Systolic blood pressure - mmHg | 125.91 (17.01) | 125.73 (16.95) |
| Diastolic blood pressure - mmHg | 77.53 (10.51) | 77.54 (10.43) |
| Antihypertensive medication - n (%) | 5,768.0 (19.1) | 11,167.6 (18.6) |
| Cholesterol-lowering medication - n (%) | 2,297.0 (7.6) | 4,513.7 (7.5) |
| Diabetes medication - n (%) | 1,074.0 (3.6) | 2,422.5 (4.0) |
| Total cholesterol - mmol/L | 5.49 (1.05) | 5.45 (1.06) |
| HDL cholesterol - mmol/L | 1.63 (0.50) | 1.59 (0.49) |
| LDL cholesterol - mmol/L | 3.44 (0.97) | 3.42 (0.97) |
| Triglycerides - mmol/L | 1.25 (0.84) | 1.29 (0.92) |
| Glucose - mmol/L | 5.77 (1.13) | 5.81 (1.20) |
| High-sensitivity C-reactive protein - mg/L | 2.14 (4.34) | 2.23 (4.61) |
| HbA1c - mmol/mol | 36.58 (6.48) | 36.90 (7.05) |
| Estimated GFR - ml/min/1.73 m^2^ | 85.10 (12.09) | 85.97 (12.23) |
| Framingham risk score - % | 11.20 (8.50) | 11.39 (8.70) |
| Personal history of myocardial infarction - n (%) | 537.0 (1.8) | 1,191.5 (2.0) |
| Previous PCI/CABG^b^ - n (%) | 556.0 (1.9) | 1,222.1 (2.1) |
| Personal history of stroke - n (%) | 465.0 (1.5) | 965.3 (1.6) |
| Personal history of peripheral artery disease (%) | 162.0 (0.5) | 362.3 (0.6) |
| Family history of premature myocardial infarction - n (%) | 2,012.0 (6.7) | 3,946.8 (6.6) |
| Family history of premature stroke - n (%) | 1,778.0 (5.9) | 3,650.8 (6.1) |
| Alcohol consumption, harmful drinking habits (AUDIT score >= 16) - n (%) | 414.0 (1.4) | 934.1 (1.6) |
| Chronic airflow limitation (FEV1/FVC<0.7) - n (%) | 2,829.0 (9.5) | 5,777.9 (9.8) |
| Wheeze - n (%) | 2,135.0 (7.1) | 4,870.6 (8.1) |
| Chronic bronchitis - n (%) | 89.0 (0.3) | 216.7 (0.4) |
| Dyspnea - n (%) | 2,874.0 (9.5) | 6,966.8 (11.6) |
| New/increased medication for airway problems (last 12 months) - n (%) | 530.0 (1.8) | 1,083.2 (1.8) |

^a Inferred using weight based on propensity scores for participation from a logistic regression model with individual-level sociodemographics, neighborhood-level sociodemographics, site and their two-way interactions as predictors.^

^b PCI, percutaneous coronary intervention; CABG, coronary artery bypass grafting.^

# Mathematical derivations

## Method for inferring the characteristics of non-participants in Table 1

We here detail the method used to infer the characteristics of non-participants in Table 1. With access to data on the participants and the population, the average characteristics of non-participants can be inferred using the law of total probability (for binary and categorical variables) and the total of total expectation (for continuous variables). To see this, let *Z* denote some characteristic and *S* be a binary participation indicator. The law of total probability implies that

|  | $P\left( Z \right)=P\left( Z \vert S=1 \right)P\left( S=1 \right)+P\left( Z \vert S=0 \right)P(S=0)$ | (A.1) |
| --- | --- | --- |

Now, solving for $P\left( Z | S=0 \right)$, we get the expression that can be used to infer the proportions of binary variables (or categories of categorical variables) among non-participants:

|  | $P\left( Z \vert S=0 \right)=\left\{ P\left( Z \right)-P(Z\vert S=1)P\left( S=1 \right) \right\}/{P(S=0)}$ | (A.2) |
| --- | --- | --- |

We proceed in the same manner for continuous variables using the law of total expectation:

|  | $E\left[ Z \right]= E\left[ Z \vert S=1 \right]P\left( S=1 \right)+E\left[ Z \vert S=0 \right]P(S=0)$ | (A.3) |
| --- | --- | --- |

Again, solving for $\left[ Z | S=0 \right]$ yields

|  | $E\left[ Z \vert S=0 \right]=\left\{ E\left[ Z \right]-E\left[ Z \vert S=1 \right]P\left( S=1 \right) \right\}/{P\left( S=0 \right)}.$ | (A.4) |
| --- | --- | --- |

## Method for calculating the AUC for classification of participants and non-participants in data where non-participants are not observed

To display the ability of our participation models to distinguish between participants and non-participants, we constructed histograms of predicted participation probabilities (**Figure S2**) and calculated the area under the ROC curve (AUC). A complicating issue was that non-participants could not be directly identified in the data. Hence, exploiting the stacked dataset would not allow us to distinguish between participants and non-participants but only between participants and individuals in the full target population To address this issue, we use an approach proposed by Nilsson et al. [1] The following explanation follows their description closely.

To construct the histograms and estimate the AUC for the model’s ability to distinguish between participants and non-participants, we needed to estimate participation probabilities for the unobserved population of non-participants. We obtained these via the law of total probability, exploiting predicted participation probabilities in the target population and the participant sample. Specifically, the law of total probability implies the following equation for the shares of individuals in different intervals with respect to participation probability:

|  | $P\left( a\leq Q<b \right)=P\left( a\leq Q<b \vert C=1 \right)*P\left( C=1 \right)+P\left( a\leq Q<b \vert C=0 \right)*P\left( C=0 \right)$ | (B.1) |
| --- | --- | --- |

Here, $Q$ is an individual’s probability of being a participant and $C$ is actual participation. $a$ and $b$ are bounds of an interval in the histogram. $Q$ was obtained from a logistic regression applied to the combined sample of participants and the target population, as described in the main text. For each interval, the left-hand side of the equation was identified from the target population and the first factor on the right-hand side from the participant sample. The corresponding quantity for non-participants was then obtained by rewriting the equation:

|  | $P\left( a\leq Q<b \vert C=0 \right)=\frac{P\left( a\leq Q<b \right)-P\left( a\leq Q<b \vert C=1 \right)*P\left( C=1 \right)}{P\left( C=0 \right)}$ | (B.2) |
| --- | --- | --- |

Using equation (B.2), a quasi-population of non-participants was constructed, which was used for the construction of the histogram of participation probabilities in **Figure S2** and to calculate the AUC so that it appropriately measures the ability of the participation models to classify participants and non-participants.

**References**

[1] Nilsson A, Bonander C, Strömberg U et al. Reweighting a Swedish health questionnaire survey using extensive population register and self-reported data for assessing and improving the validity of longitudinal associations. PLOS ONE 2021;16:e0253969.
