# Supplementary material for: The value of combining individual and small area sociodemographic data for assessing and handling selective participation in cohort studies: Evidence from the Swedish CardioPulmonary bioImage Study
Source: PLoS One. 2022 Mar 8;17(3):e0265088. doi: 10.1371/journal.pone.0265088 (PMC8903292; doi:10.1371/journal.pone.0265088)
Supplement: Attachment — Submitted filename: Response to reviewers.docx [file pone.0265088.s002.docx]

Table 1. Editorial comments

| Comment | Response |
| --- | --- |
| 1. When submitting your revision, we need you to address these additional requirements.  Please ensure that your manuscript meets PLOS ONE's style requirements, including those for file naming. | We have reviewed PLOS ONE’s style requirements and made corresponding changes to heading font size, tables, and the title page. |
| 2. Thank you for stating the following in the Funding Section of your manuscript:  “The study presented in this paper was funded by research grants from Swedish Research Council for Health, Working life and Welfare (Forte, grant no. 2017-00414; 2020-00962) and the Swedish Research Council (VR, grant no. 2019-00198). SCAPIS also received external funding from Swedish Heart-Lung Foundation, Knut and Alice Wallenberg Foundation (grant no. 2014-0047), Swedish Research Council (grant no. 822-2013-2000) and VINNOVA (Sweden’s Innovation agency, grant no. 2012-04476), and internal funding from University of Gothenburg and Sahlgrenska University Hospital, Karolinska Institutet and Stockholm county council, Linköping University and University Hospital, Lund University and Skåne University Hospital, Umeå University and University Hospital, Uppsala University and University Hospital (grant numbers not applicable for internal sources of funding). The funders had no role in study design, data collection and analysis, decision to publish, or preparation of the manuscript.”  We note that you have provided funding information that is not currently declared in your Funding Statement. However, funding information should not appear in the Acknowledgments section or other areas of your manuscript. We will only publish funding information present in the Funding Statement section of the online submission form.  Please remove any funding-related text from the manuscript and let us know how you would like to update your Funding Statement. Currently, your Funding Statement reads as follows:  “The study presented in this paper was funded by research grants from Swedish Research Council for Health, Working life and Welfare (Forte, [www.forte.se](http://www.forte.se), grant no. 2017-00414; 2020-00962) and the Swedish Research Council (VR, [www.vetenskapsradet.se](http://www.vetenskapsradet.se), grant no. 2019-00198). SCAPIS also received external funding from Swedish Heart-Lung Foundation ([www.hjart-lungfonden.se](http://www.hjart-lungfonden.se), grant no. not available), Knut and Alice Wallenberg Foundation ([www.kaw.wallenberg.org](http://www.kaw.wallenberg.org), grant no. 2014-0047), Swedish Research Council ([www.vetenskapsradet.se](http://www.vetenskapsradet.se), grant no. 822-2013-2000) and VINNOVA (Sweden’s Innovation agency, [www.vinnova.se](http://www.vinnova.se),  grant no. 2012-04476), and internal funding from University of Gothenburg and Sahlgrenska University Hospital, Karolinska Institutet and Stockholm county council, Linköping University and University Hospital, Lund University and Skåne University Hospital, Umeå University and University Hospital, Uppsala University and University Hospital (grant numbers not applicable for internal sources of funding). The funders had no role in study design, data collection and analysis, decision to publish, or preparation of the manuscript.”  Please include your amended statements within your cover letter; we will change the online submission form on your behalf. | We have now removed the funding statement from the paper. Please note that the Funding statements in the paper and the statement in the online submission system contain identical information about funding sources. The only difference is that we provided websites (for applicable funding sources) in the online system. The current funding statement is therefore correct and should therefore not require amendment. |
| 3. In your Data Availability statement, you have not specified where the minimal data set underlying the results described in your manuscript can be found. PLOS defines a study's minimal data set as the underlying data used to reach the conclusions drawn in the manuscript and any additional data required to replicate the reported study findings in their entirety. All PLOS journals require that the minimal data set be made fully available. For more information about our data policy, please see <http://journals.plos.org/plosone/s/data-availability>.  Upon re-submitting your revised manuscript, please upload your study’s minimal underlying data set as either Supporting Information files or to a stable, public repository and include the relevant URLs, DOIs, or accession numbers within your revised cover letter. For a list of acceptable repositories, please see [http://journals.plos.org/plosone/s/data-availability#loc-recommended-repositories](http://journals.plos.org/plosone/s/data-availability#loc-recommended-repositories). Any potentially identifying patient information must be fully anonymized.  Important: If there are ethical or legal restrictions to sharing your data publicly, please explain these restrictions in detail. Please see our guidelines for more information on what we consider unacceptable restrictions to publicly sharing data: [http://journals.plos.org/plosone/s/data-availability#loc-unacceptable-data-access-restrictions](http://journals.plos.org/plosone/s/data-availability#loc-unacceptable-data-access-restrictions). Note that it is not acceptable for the authors to be the sole named individuals responsible for ensuring data access.  We will update your Data Availability statement to reflect the information you provide in your cover letter. | There are legal restrictions in our case. We cannot upload the data, even anonymous versions of it. We have amended the Data availability statement to explain the restrictions further.  The amended version reads (new text highlighted):  **Data availability statement**  The Regional Ethics Committee in Umeå has approved the study according to the Swedish Ethical Review Act (2003:460) regarding the ethical review of research involving humans (diary number 2010-228-31M, with addendum 2011-02-21, for SCAPIS and 2016-511-31 for the linkage of register data to SCAPIS participants). As dictated by the ethical body that approved the study and the promise to participants in their informed consent, the research data collected in the present study cannot be shared publicly as the data contain potentially identifying and sensitive personal data according to article 9 the General Data Protection Regulation (EU 2016/679), and public availability would compromise participant privacy. The General Data Protection Regulation (EU 2016/679) also classifies de-identified versions of sensitive data that are sufficiently detailed to allow for re-identification as sensitive personal information. According to Swedish law (Law 2003:460 for ethical review of research involving humans), ethical permission is required to process such data. In accordance with Swedish legislation, the data required to replicate our study can and will be made available to researchers who meet the criteria for access to confidential data, which includes obtaining their own ethics approval from the Swedish Ethical Review Authority (email: [registrator@etikprovning.se](mailto:registrator@etikprovning.se); website: <https://etikprovningsmyndigheten.se>). Data applications can then be made by contacting SCAPIS (email: [scapis@scapis.org](mailto:scapis@scapis.org); website: <https://www.scapis.org/data-access/>). |
| 4. We note that you have indicated that data from this study are available upon request. PLOS only allows data to be available upon request if there are legal or ethical restrictions on sharing data publicly. For more information on unacceptable data access restrictions, please see [http://journals.plos.org/plosone/s/data-availability#loc-unacceptable-data-access-restrictions](http://journals.plos.org/plosone/s/data-availability#loc-unacceptable-data-access-restrictions).  In your revised cover letter, please address the following prompts:  a) If there are ethical or legal restrictions on sharing a de-identified data set, please explain them in detail (e.g., data contain potentially sensitive information, data are owned by a third-party organization, etc.) and who has imposed them (e.g., an ethics committee). Please also provide contact information for a data access committee, ethics committee, or other institutional body to which data requests may be sent.  b) If there are no restrictions, please upload the minimal anonymized data set necessary to replicate your study findings as either Supporting Information files or to a stable, public repository and provide us with the relevant URLs, DOIs, or accession numbers. For a list of acceptable repositories, please see [http://journals.plos.org/plosone/s/data-availability#loc-recommended-repositories](http://journals.plos.org/plosone/s/data-availability#loc-recommended-repositories).  We will update your Data Availability statement on your behalf to reflect the information you provide. | Our previous data availability statement included information about legal restrictions with reference to European legislation (GDPR). We have now further clarified why we cannot share the data publicly, as even anonymized versions would be classified as sensitive personal information and require ethical approval to be processed according to European and Swedish legislation. However, Swedish universities are required to share the data with researchers with ethical permission from a Swedish ethical review board who wish to access it. |
| 5. Please note that in order to use the direct billing option the corresponding author must be affiliated with the chosen institute. Please either amend your manuscript to change the affiliation or corresponding author, or email us at [plosone@plos.org](mailto:plosone@plos.org) with a request to remove this option. | The corresponding author is affiliated with the chosen institute. We have now removed titles from each name in the title page information according to the style guide; perhaps this was the reason why the author was not identified as being affiliated with the chosen institute in the previous version? If not, please advise. |

Table 2. Reviewer #1

| Comment | Response | Changes made |
| --- | --- | --- |
| The manuscript presents a useful approach of improving the accuracy of the SCAPIS participation model by combining individual and small area sociodemographic data. Reweighting the study participants based on this model led to larger changes in cardiopulmonary risk factor distributions than using either data source alone. The combination of individual and area-level data shows a potential improvement of the assessment and handling of selective participation in cohort studies. | We thank reviewer #1 for the positive feedback and for taking the time to review our manuscript. |  |

Table 3. Reviewer #2

| Comment | Response | Changes made |
| --- | --- | --- |
| The authors have addressed an important issue in an intelligible fashion, written in standard English. Moreover, the manuscript is technically sound, and the data support the conclusions. The statistical analysis has been performed appropriately and rigorously. | We thank reviewer #2 for the positive feedback and for taking the time to review our manuscript. |  |

Table 4. Reviewer #3

| Comment | Response | Changes made |
| --- | --- | --- |
| The research presents a new method for evaluating and dealing with selective participation, which could lead to the enhancement of the results obtained, but attention should be paid to the following issues in order to enhance the results of this study: | We thank reviewer #3 for taking the time to review our manuscript and providing critical feedback. |  |
| _ There is a need to rewrite the abstract section according to the way used in POLS ONE, which is in the form of a single unstructured continuous paragraph; | We have not been able to find this instruction in PLOS ONE’s submission guidelines, and there are many examples of papers in PLOS ONE that have used structured abstracts. We believe that both versions are fine. | We have maintained the structured abstract. |
| - The researchers did not fully explain why they made restrictions to data availability, although it is possible to provide this data after concealing the information that may lead to the disclosure of the personal identity of the participants; | We agree with the first part of this statement, but disagree with the last. According to Swedish legislation, individual-level health data is considered personal information, even if identifiers are removed. The legal rationale is that backward identification may be possible by combining rich information. Legally, we can only share the data required to replicate our analyses with other researchers after they have obtained permission to process the data from a Swedish ethical review board.  We can legally upload some of the data (e.g., the data files containing basic sociodemographics on the population). But those data are not sufficient to replicate any of our analyses, as the analyses also require individual-level data from the SCAPIS participants, which we cannot share without legal permission. | We have clarified the legal restrictions further in the Data availability statement. |
| The introduction of the draft includes some statistical terms that need further clarification, and no explanatory idea or clarification was given about the SCAPIS study or any other studies that were conducted on this subject. All these issues can help to reach the rationale and justification for doing this study. | Thank you for this useful comment. We agree that adding these points can help improve the introduction to the manuscript. | Firstly, we have clarified or removed unnecessary statistical terms from the introduction.  Secondly, we moved the information about SCAPIS (previously at the start of the methods section). We added references to previous work (previously in the discussion) to the introduction section to further clarify the study's rationale.  Thirdly, given the above additions, we also revised the final paragraph of the introduction (containing the aims) to justify the study further. |
| The method section is better to divide it into subsections and explain in a clearer way the method of work. Some other important points should be added such as the method of sampling, data collection, exclusion criteria and the period and duration of the study. It is better to put all tables in the results section. At the end of the method, there should be a subsection for statistical analysis; | Thank you for pointing this out. We agree with everything in this comment except for the position of the table; we believe that it fits better in the methods section as it is important for explaining the target population and recruitment period (and how they vary by site). It could also fit in the results, but PLOS ONE requires that tables are placed directly after the paragraph/sentence where they are first mentioned. | We have divided the methods section into smaller subsections. We have also added details to clarify the sampling method, data collection, and study period. |
| The discussion section did not discuss the results with studies that used the same or comparable method in work, which may affect the generalizability of this method and make it like a pilot project | We agree but have not found any similar work to discuss in detail. Updated searches on PubMed and a new screening for papers referencing key methodological literature on generalizability did not yield any additional studies at the time of revision. | We have updated the introduction, now mentioning the (lack of) previous work on the topic more clearly. We have also added a sentence to the second paragraph of the discussion section to highlight the importance of additional work to replicate and confirm our findings. |
| There are some linguistic errors and grammatical problems that affect the structure and context of phrases and sentences. it greatly affected the clarity of the research material. | We agree that linguistic issues are very important for clarity. | We re-assessed the text to improve clarity and structure and fix grammatical issues. |

Table 5. Reviewer #4

| Comment | Response | Changes made |
| --- | --- | --- |
| The population-based Swedish Cardio Pulmonary bioImage Study (SCAPIS) is a prospective observational study of a randomly selected sample from the general population and baseline clinical examinations were carried out between 2013 and 2018. The purpose was to improve the risk prediction of cardiopulmonary diseases in the general population by obtaining information on underlying disease mechanisms with a view to better prevention and treatment of CVD, COPD and associated metabolic diseases. This present study is quite an excellent informative one where external individual and neighborhood (small area units) level register data on SCAPIS participants were combined with data from a random sample of their target population to form a stacked dataset. This study finds that using a combination of individual and neighborhood level characteristics improved the accuracy of the participation probabilities for the SCAPIS (predicting participation in SCAPIS) better than using either dataset alone. Furthermore, reweighting the SCAPIS participants using this combined model, led to more marked changes in the cardiopulmonary risk factor distributions at baseline although this change was found to be more meaningful in only one risk factor : self-reported frequency of alcohol consumption.  One of the main strengths of this paper is the inclusion of neighborhood level data at baseline selection of study participants and testing the value of the combined dataset in relation to the individual only and neighborhood only datasets. Thus selection effects at all levels were assessed. Additionally, some of the acknowledged methods for adjusting for selection bias in cohort and other studies were applied in this study. These are inverse probability weighting, controlling for covariates associated with selection and bias analysis.  However, a few clarifications are needed. If these are addressed, I believe the authors would have satisfied the publication criteria for PLOS ONE. These clarifications are the following: | We thank reviewer #4 for taking the time to review our manuscript and providing critical feedback. |  |
| (Introduction)  Is there evidence from literature that a similar study, testing these sets of variables (individual only, neighborhood only and combined) (howbeit for another set of risk factors or other diseases) has been carried out? If so, could the authors please comment on the findings of such studies and if none was found; state so. | We agree that the introduction benefits from adding this information to motivate our study clearly. We are only aware of one study investigating the use of aggregate data versus individual data to handle sample selection. In the previous version, we mentioned it very briefly in the discussion. Updated searches on PubMed and a new screening for papers referencing key methodological literature on generalizability did not yield any additional studies at the time of revision. | We now mention one identified related study in the introduction, their findings, and state how our focus differs from theirs. |
| (Methods)  The authors did not indicate when this present study was carried out and the duration. | Thank you for pointing out our omission. Our previous version only contained the study period for SCAPIS, but we failed to explicitly mention that the study period for the register data was the same period at each site. | We have now detailed the study period for the present study more clearly in the methods section. |
| The authors might consider mentioning the relationship between one neighborhood in line 99 (where approximately 280 individuals live) and one DeSO in Sweden. Otherwise the following phrase in line 97 to 98 would be confusing- “In this paper, we refer to these area units as neighborhoods” -since the randomly selected target population was way above 280. This is for the benefit of readers not familiar with this Swedish system. | We agree that the context was difficult to follow as presented. | We have provided additional context and information on DeSOs in conjunction with the statement. |
| It appears that the following elements were omitted from the model with area level data only in line 126 (but they were mentioned earlier on in the paper): high income, Nordic origin, unemployed working age. | We thank the reviewer for noting our omission of the following information: (i) unemployed working age is included in the model, but we failed to mention it on line 126; (ii) we omitted high income and Nordic origin from the model to avoid perfect collinearity with the other categories in the aggregate income and country of birth variables but did not mention why in the paper. | We have now added point (i) to the list of variables in the methods section and clarified why we omitted the variables in point (ii) from the model in a new sentence directly following this information. We also slightly restructured the paragraph containing this information to improve the flow given this new sentence. |
